# Case 6 / 2016 - Heart Failure in a 23-Year-Old Male with a History of Illicit Drug Use

**DOI:** 10.5935/abc.20160189

**Published:** 2016-12

**Authors:** Fernando Faglioni Ribas, Paulo Sampaio Gutierrez

**Affiliations:** Instituto do Coração (InCor) HC-FMUSP, São Paulo, SP - Brazil

**Keywords:** Heart Failure / physiopathology, Street Drugs, Drug Users, Young Adult, Diagnostic Imaging

Male patient, 23 years-old, sought medical treatment for malaise, nausea, vomiting, and retrosternal burning pain for three days (09/25/2013). Two weeks before seeking medical attention, the patient was diagnosed with a heart disease after an evaluation done one month before his complaint of malaise, for a research of dyspnea during physical effort with progressive worsening. He stated to have been a cocaine user in the past, but had been clean for seven years.

Transthoracic two-dimensional echocardiogram (09/11/2013) showed: left atrium diameter 58 mm; left ventricle diameters (diast./syst.) 81 mm/ 72 mm, LVEF= 24%; accentuated diffuse ventricular hypokinesis; restrictive filling pattern; moderate to severe mitral insufficiency. The patient was prescribed: enalapril 1 omg, furosemide 40 mg, spironolactone 25 mg, and carvedilol 6.25 mg daily.

Lab exams (09/19/2013) revealed: urea 48 mg/dL, creatinine 1.82 mg/dL, sodium 140 mEq/L, potassium 4.8 mEq/L.

During the physical exam (09/25/2013), the patient presented regular overall condition, acyanotic, afebrile, and hydrated; heart rate was 92 bpm; blood pressure was 80x60 mmHg, arterial saturation 98%; pulmonary auscultation was normal; heart auscultation showed the presence of third sound and regurgitant systolic murmur +++/6+ in mitral area; abdominal exam was normal, and there was no edema in the lower limbs.

Electrocardiogram showed overload of the left chamber.

Lab exams (09/25/2013) revealed: CKMB 1.61 ng/mL, troponin I 0.447 ng/mL, urea 60 mg/dL, creatinine 2 mg/dL, C-reactive protein 2.65 mg/L, sodium 139 mEq/L, potassium 4.3 mEq/L, PT (INR) 1.3, PTT (rel) 0.87, hemoglobin 16.8 g/dL, hematocrit 49%, leukocytes 9100/mm^3^ (61% neutrophils, 1% eosinophils, 1% basophils, 30% lymphocytes, and 7% monocytes), platelets 286000/mm^3^.

Toxicology screen (results obtained on October 10^th^) was positive for benzodiazepine and ecstasy, negative for amphetamines, methamphetamines, cocaine, opioids, barbiturates, and marijuana.

Chest X-Ray (09/29/2013) showed pronounced cardiomegaly with lung fields without condensation (Fig. 1)

**Figure 1:**
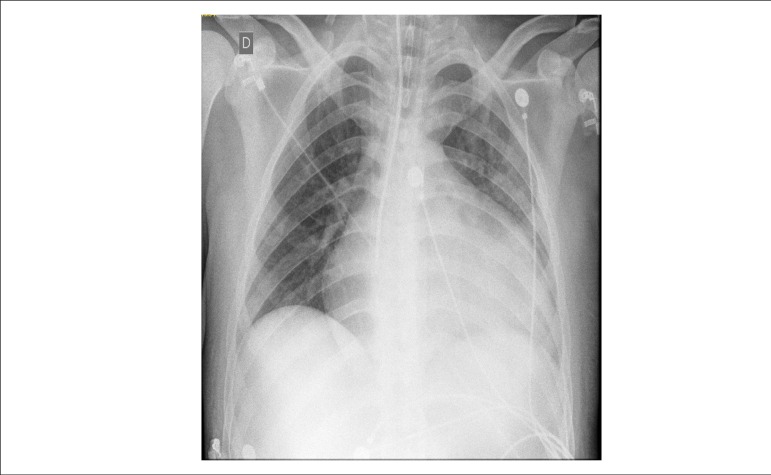
Chest X-Ray. Severe cardiomegaly, free lung fields.

A new echocardiographic evaluation (09/27/2013) showed aortic diameter of 27 mm, left atrium diameter of 57 mm, mean right ventricle diameter of 31 mm, left ventricle diameters (diast./syst.) 80/73, ejection fraction 20%, and septum and posterior wall thickness of 9 mm. The left ventricle was diffusely hypokinetic, more pronounced in the inferior wall; there was accentuated mitral insufficiency by failure of coaptation of cusps, as well as indirect sings of pulmonary hypertension by the movement analysis of the sigmoid of the pulmonary valve; pericardium was normal. (Figures 2, 3, and 4)

**Figure 2:**
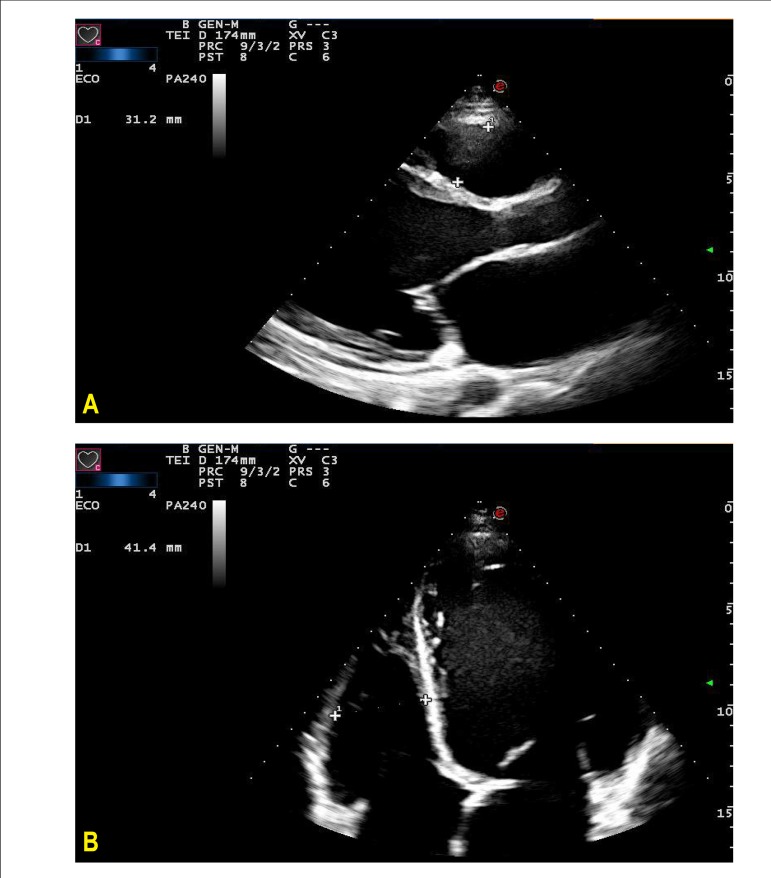
Echocardiogram. A) Longitudinal parasternal view. Enlargement of left ventricle and atrium; B) Apical four chamber view. Enlargement of the ventricle with auto contrast in apical position.

**Figure 3:**
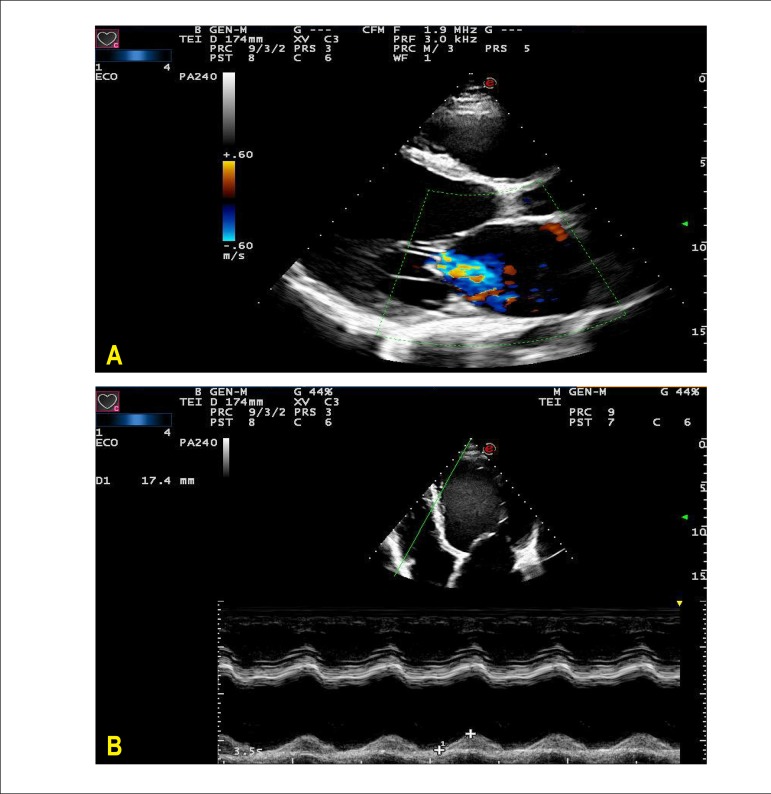
Echocardiogram. A) Longitudinal parasternal view with Doppler. Severe mitral insufficiency. B) Apical four chamber view and one-dimensional echocardiogram of the left ventricle demonstrating paradoxical movement of the interventricular septum.

**Figure 4:**
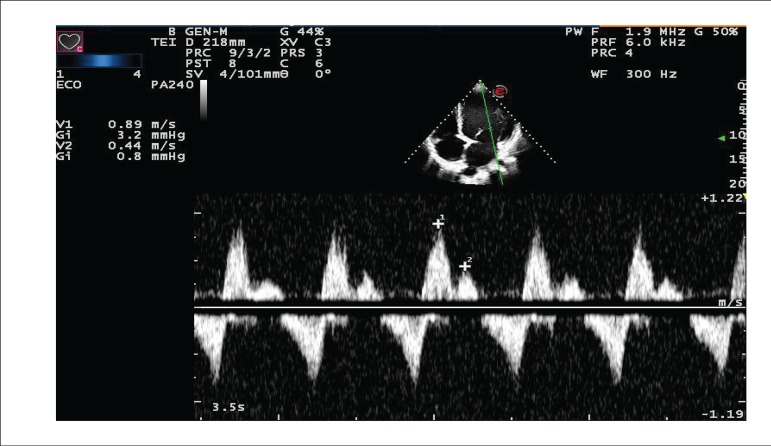
Echocardiogram. Restrictive ventricular filling.

MRI (09/27/2013) showed: right atrium with normal dimensions, right ventricle with pronounced dilatation (indexed end diastolic volume = 131 mL/m^2^, indexed end systolic volume = 97 mL/m^2^) with depressed systolic function (EF=25%), and accentuated enlargement of the left atrium and left ventricle, diameters (diast./syst. 96/83 mm and indexed end diastolic volume = 282 mL/m^2^, indexed end systolic volume = 218 mL/m^2^), ejection fraction 23%, basal, mean and apical septal hypokinesis, inferior akinesia and akinesia in mid-basal and inferolateral segments. There was late mesocardial enhancement in all the mid-basal and apical septal walls and in the subepicardial of the mid-basal and inferolateral segments. The findings were considered of a pattern non-secondary to ischemic event. Septum thickness was 9 mm and lateral wall thickness was 4 mm. There was also pericardial effusion with no filling restrictions. (Figure 5)

**Figure 5:**
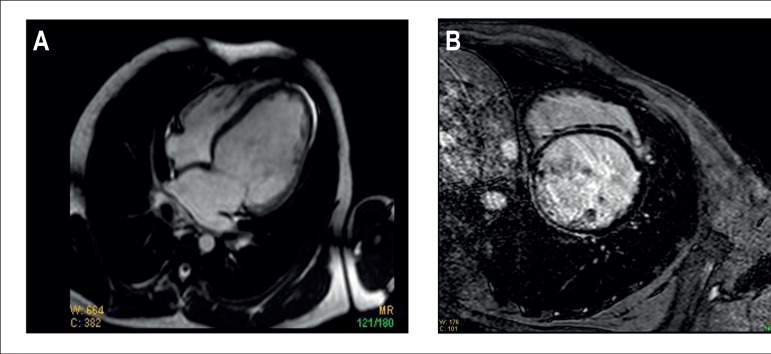
A) Cardiac MRI. Dilated left ventricle and atrium, presence of pericardial effusion, with no diastolic restriction. B) Cardiac MRI. Presence of late mesocardial enhancement in the septum and transmural in the inferior wall.

Abdominal ultrasound (10/02/2016) showed hepatomegaly of the right lobe, ectasia of the vena cava and hepatic veins, gallbladder with sludge, normal pancreas, spleen with increased volume, topical kidneys, preserved dimensions (right kidney 10.5 cm and left kidney 11.5 cm, preserved thickness and bilateral hyperechogenicity.

Initially, the patient responded well to treatment, but with a lot of agitation and anxiety. However, he later progressed with a worsening of the dyspnea and hypotension attributed to a probable infection of pulmonary focus, requiring the use of vasopressor amines at maximum doses, orotracheal intubation for mechanical ventilation and passage of the intra-aortic balloon. He was initially treated with piperacillin and tazobactam, with therapeutic amplification to vancomycin and meropenem and colistin and fluconazole on October 15^th^. He progressed to dysfunction of multiple organs, including kidney failure requiring continuous hemodialysis.

## Table 1. Laboratory evolution

On October 15^th^, there was a need for a progressive increase of vasopressors. Dialysis was recommended for kidney insufficiency and hyperkalemia, but the patient did not tolerate it, and, after two hours, dialysis was stopped on the night of October 17^th^ 2013, and he received an association of vasopressors as well as a correction of metabolic disorders. Despite high doses of vasoactive drugs, with noradrenaline 3.0 mcg/kg/min, vasopressin 0.04 UI/min, dobutamine 20 mcg/kg/min, intra-aortic balloon 1:1, the patient went into cardiorespiratory arrest in pulseless electrical activity, and died at 00:45 on October 18^th^, 2013.

## Clinical aspects

This is the case of a young male patient with dilated cardiomyopathy of unknown etiology. Didactically, we can investigate heart failure seeking anatomical and functional alterations.^1^ From the anatomical perspective, the syndrome can stem from alterations in the myocardium, endomyocardium, or pericardium. From the functional perspective, it can be caused by conduction disorders (tachyarrhythmia and bradyarrhythmia) or load disturbances (volume overload, systemic hypertension, valve and structural diseases). Myocardial injuries are the most the common causes. In this stratum lies the ischemic etiology. Although there are no classic risk factors for stable coronary disease in this case, ischemic etiology must be considered due to the history of illicit drug use. In young individuals, in the context of acute coronary syndromes, there are mechanisms other than classic coronary plaques, such as vasospasm, intracoronary thrombosis^2^ or spontaneous dissection.^3^ Other myocardial injuries are not infrequent as a cause for ventricular dilatation, by myocarditis (infectious or not), direct toxicity (use of recreational drugs), or genetic dysfunctions (sporadic or familial). Guidelines for cardiac failure also contemplate infiltrative diseases (by malignancy or not) or hormonal and nutritional metabolic alterations.^1,2^

From the epidemiological perspective, because this was a young patient with no history of hypertension, dyslipidemia, diabetes, or smoking, the possibility of chronic coronary disease is slim. We must keep in mind that the clinical history is of a subacute disease, with classic symptoms of heart failure that started only one month before, with a quick decline of ventricular function and patient stability. Any case of new cardiac failure with major dysfunctions must include myocarditis among its differential diagnoses.^4^ The patient's history of illicit drug stands out, and brings hypotheses of direct damage to the myocardium due to the drugs, which provoked coronary dysfunctions, especially microcirculation. There is no history of previous viral conditions or family history of heart disease.

The physical exam is not sufficiently detailed to converge the etiological diagnostic hypotheses, but it proves that there is heart failure due to the presence of B3, one of the major criteria of Framingham.^5^ In the lab exams, the normality of plasma level of sodium, even without adequate treatment time, can corroborate the subacute character of the disease.

In the complementary investigation, electrocardiogram shows an overload of the left chambers, not a typical pattern of any etiology. It is important to remember that in infiltrative diseases such as amyloidosis, for example, there is a reduction in QRS voltage,^6^ which does not occur in this case. There are also no alterations related to previously described ischemia, such as inactive areas. Even though the report does not mention serology for Chagas disease, it is known that Chagas patients who progress with a worse diagnosis, usually present alterations in the ECG with right bundle branch block and blockage of the anterior superior division of the left branch.^7^

Echocardiogram confirms the diagnostic hypothesis of heart failure, showing an extremely dilated left ventricle, with segmental dysfunction in the inferior wall and important systolic ventricular dysfunction. There are no alterations described in the endomyocardium, which rules out restriction by endomyocardial fibrosis, common in tropical countries. There are also no alterations described in the pericardium, or data that suggest constriction. We also ruled out primary valve alterations. Thin wall thickness does not match infiltrative and restrictive diseases, and there were no restrictive diastolic patterns. Moreover, these diseases rarely come with systolic dysfunction, and diastolic dysfunction is usually predominant.^6^ Thus, extreme dilatation with segmental dysfunction suggests myocardial damage, be it by ischemia, inflammation, or even primary dilated cardiomyopathy (DCM).

MRI findings confirm myocardial disease, with no anatomic alterations of the pericardium or endomyocardium, and show late mesocardial enhancement in practically the entire septal wall and late subepicardial enhancement in the inferolateral wall, in the mid and basal segments. The inferior wall, akinetic and with late enhancement, is extremely thin, hindering characterization regarding a transmural pattern versus mesocardial pattern. Late enhancement in the MRI represents a persistency in the impregnation by contrast, and suggest the presence of areas of myocardial fibroses. When this scar is transmural, that is, covers the whole myocardial wall thickness, we describe it as an ischemic pattern of enhancement. Transmural enhancement can also occur by myocarditis, but we cannot rule out ischemia with this pattern. On the other hand, non-transmural enhancement (mesocardial or subepicardial) does suggest another etiology, such as myocarditis or non-ischemic cardiomyopathies, considering ischemic processes occur from the endocardial to the epicardium.^6^ With inflammatory diseases such as myocarditis, Lake Louise criteria have been recently published, which have increased the accuracy of the diagnostic exam. Together with late enhancement, we have another two criteria: T2 weighting and early enhancement, both of which evidenced acute inflammation and muscle wall edema.^8^ Late enhancement in isolation, as in this case, reduces exam specificity to 46% in myocarditis, accepting an ample differential diagnosis.^8^ Another condition that often presents late mesocardial enhancement in wide areas is primary DCM itself, representing a poor prognosis for this disease.^6^ Regarding the doubt faced by the team in relation to the inferior wall, it was not possible to establish a precise diagnosis with this exam. Due to the use of drugs, the patient may have presents inferior wall ischemia, and progressed with DCM by direct toxicity of other myocardial regions. The patient may also have had DCM from a previous viral myocarditis or idiopathic DCM and presents the inferior alteration due to the extreme thinning of this regions.

**Table 1:** Lab exams

Exam	30 set	01 out 2013	15 out	17 out
Hemoglobin (g/dL)	12.6	13	10.3	8.7
Hematocrit (%)	38%	42	36	29
Leukocytes (/mm^3^)	6880	8590	17770	31440
Segmented (%)	69	75	68	57%
Metamyelocytes (%)			1	1
Rod cells (%)			18	22
Eosinophils (%)	1	1	1	1
Linocuts (%)	30	18	9	16
Monocytes (%)	7	5	2	3
Platelets/mm^3^	286000	168000	291000	228000
Sodium (mEq/L)	136	132	146	144
Potassium (mEq/L)	3.5	3.4	6.6	4.2
CK MB (ng/L)	0.83			
Troponin I (ng/L)	0.406			
Urea (mg/dL)	48	53	120	122
Creatinine (mg/dL)	1.85	1.91	3.62	2.01
PCR (mg/L)		28.8	136.25	119.59
Arterial Lactate (mg/dL)	11	11	53	99
Arterial gasometry				
pH			7.19	7.15
pCO2 (mm Hg)			45.7	39.6
pO2 (mm Hg)			110	124
Sat. O2 (%)			98.4	98
HCO3 (mE/L)			16.8	13.2
Excess Base (mEqL)			- 10.7	- 14.2
AST (U/L)		20	366	1074
ALT (U/L)		30	64	960
Gamma GT (U/L)		118	104	206
FA (U/L)		66	84	76
Total bilirubin (m/dL)			2.51	3.24
Direct bilirubin (MG/dl)			1.26	2.24
PT (INR)		1.3	2.2	1.8
PTT (rel)		0.93	0.92	0.85
Density				1.032
Proteins (g/L)				3.31
Epithelial cells /mL				3000
Leukocytes/mL				8000
Erythrocytes/mL				23000

PCR: P reactive protein; AST: Aspartate aminotransferase; ALT: Alanine aminotransferase; Gama GT: Gamma-glutamyl transpeptidase; FA: Alkaline Phosphatase; PT: Prothrombin time; PTT: Partial thromboplastin time.

After MRI results, the assisting team obtained tox screen results: positive for ecstasy and benzodiazepines. Regarding the patient's statement of being recently clean of illicit drugs, a false positive is unlikely at this point because the blood was collected before the ingestion of most medications that may have generated crossed reactions in the results. Moreover, there are reports of considerable agitation and anxiety from the patient throughout his stay, which can represent a state of withdrawal from the previously used substances.

By conflating the several collected data, clinical history, and complementary tests, we can suppose that our differentials were restricted to ischemic/toxicologic cardiomyopathy caused by cocaine and ecstasy, leading to the differential diagnosis of viral/inflammatory myocarditis and chronic phase of primary DCM.

In a case such as this one, of subacute heart failure refractory to clinical treatment, it is recommended to perform a endomyocardial biopsy as a grade 1 recommendation and evidence level B in the Statement^9^ of the American and European Societies published in 2007.

The incredibly fast evolution of the condition made it impossible to perform an endomyocardial biopsy at that moment.

Cardiomyopathy associated to cocaine use is not yet fully understood. The incidence is under 1% of DCMs, according to a study from the John Hopkins University.^10^ In comparison to ecstasy, the cardiovascular pathophysiology related to cocaine is better described in the literature. Its most notable effect is the nora-adrenergic stimulatory action, which inhibits the reuptake of noradrenaline in the synaptic clefts.^11^ That promotes a sympathetic discharge with vasoconstriction (alpha effect), and an increase in cardiac frequency and contractility (beta effect).^11^ Coronary risks become higher because there is more oxygen consumption with a reduction of supply. Moreover, it was demonstrated, in this context, an increase in thrombotic diathesis from platelet activation, release of fibrinogen and Von Willebrand factor, as well as an increase of tissue plasminogen activator inhibitors activity.^12,13^ This combination of factors increases the chance of intracoronary thrombosis.^13^ Additionally, Wilbert-Lampen et al.^14^ have demonstrated an increased release of endothelin in cocaine users, a powerful vasoconstrictor that contributes to endothelial dysfunction in these patients. A study published in 1996 proved endothelial dysfunction in these patients as well as a worse flow response, even without the context of acute intoxication, showing that endothelial alterations in chronic users are persistent.^15^ We can thus conclude that, even without atherosclerotic plaque, microcirculation dysfunction, the potential of vasospasm and intracoronary thrombosis, and the imbalance between oxygen supply and consumption provoked by the drug allow the occurrence of acute myocardial infarction and, consequently, the appearance of ischemic cardiomyopathy.

Although classically described and very important, alterations in coronaries and microcirculation in cocaine users do not sufficiently explain cases of drug-related DCM. Some hypotheses of direct toxic damage to the myocardium include lymphocytic infiltrate, as well as an increase in intracellular levels of calcium due to the beta-adrenergic stimulus, generating necrosis of the cardiomyocyte. Recently, an Italian group demonstrated indications of the theory of oxidative stress generated by the drug, propagating the myocardial lesion, as an activity of oxidative enzymes and direct cellular damage markers.^16^

Methylenedioxymethamphetamine, known as 'ecstasy', acts in the same way as methamphetamines, except it is also linked to serotonin receptors.^17^ Most amphetamine related DCM patients are male, and the few reported cases were presented early with severe dysfunction. In one of the few studies on this theme, Yeo et al.^18^ observed an increased prevalence of methamphetamine users in a young population (<45 years old) with idiopathic DCM, generating a hypotheses that the drug may be a causal factor or an accelerator of the disease. A recent review discussed the etiology of cardiomyopathy in methamphetamine use.^19^ The variables that potentially cause dysfunctions are countless. Tachycardia, the state of hyper stress, and catecholaminergic release are included. The same pathophysiology of cocaine generates ischemia. There are also hypotheses described regarding oxidative stress, pathway activation resulting in apoptosis, an increase of calcium and intracellular free fatty acids.^19^

**(Dr. Fernando Faglioni Ribas)**

**Final comments and diagnostic hypothesis:** Assuming this was a case of DCM by a direct drug cardiotoxicity and related ischemic alterations, regardless of the substance that caused them, the prognosis with such dilatation and fibroses is limited.^5^ Unfortunately, the patient did not have a satisfactory outcome with the instituted therapeutic measures, perhaps due to precipitating infectious factors, but that probably would not result in the same way were it not for the established cardiac dysfunction. An alarming increase in the number of illicit substance users^20^ makes it pivotal that doctors have deep knowledge of the pathophysiology of these diseases, their prevention and treatment.

**(Dr. Fernando Fagliono Ribas)**

## Necropsy

**Figure 6:**
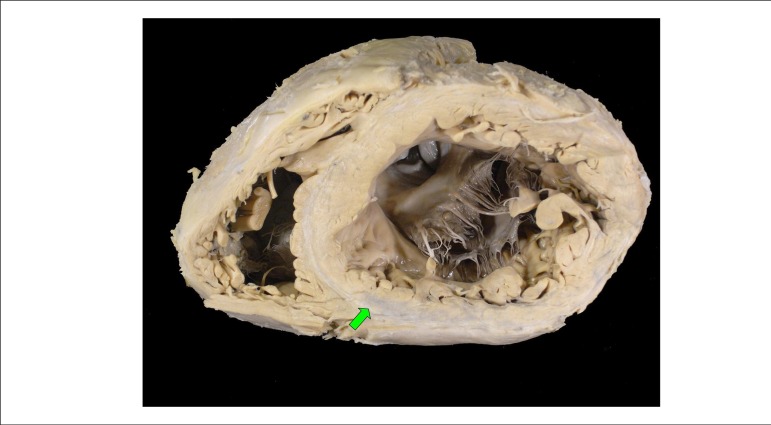
Cross section of the heart showing acute dilatation and areas of myocardial fibroses with whitish coloration; the arrow shows the largest one, in the diaphragmatic wall (posterior, inferior) of the left ventricle.

**Figure 7:**
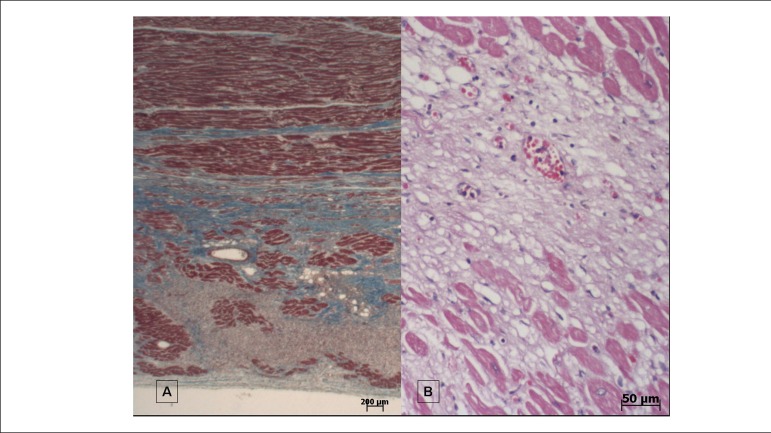
Histologic sections of the heart. A) area of fibrosis, stained in blue (Masson’s trichrome staining method, objective magnification=1x; B) microinfarction area in organization (staining by hematoxylin and eosin, objective magnification=10X).

**Figure 8:**
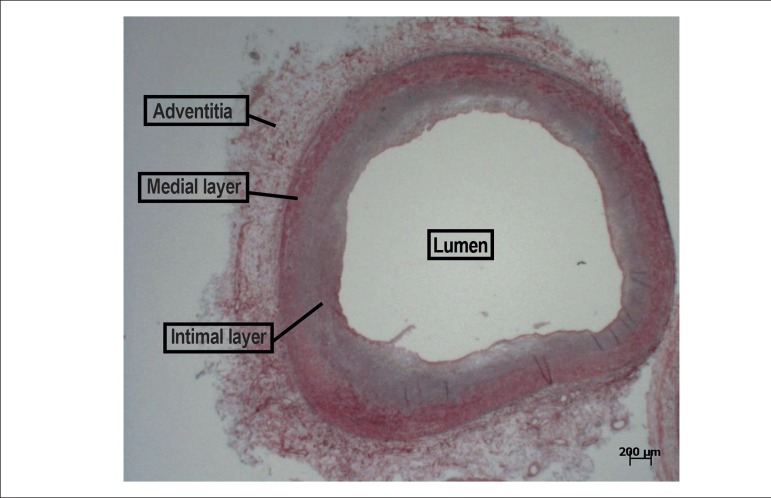
Histologic section of the coronary artery showing that the light does not present a significant level of obstruction (Movat pentachromic staining method, objectioev magnification=1X).

During the necropsy, the heart showed accentuated dilatation with hypertrophy and wall thickness close to normal (1.5 cm - normal up to 1.2 cm), that is, the so called "eccentric" hypertrophy that accompanies dilatation (Figure 6), in which there is a addition of sarcomeres in series. The heart weighed 1038 g (normal between 350 and 400 g). There was myocardial fibroses more located in the diaphragmatic wall (posterior, inferior) of the left ventricle, accompanied by other areas of fibroses in all the chamber's walls - especially in the medomural region - or by ischemic-pattern necrosis in organization, with characteristics suggestive of an approximately two-week evolution, especially in the subendocardial region (Figure 7). Coronary arteries did not show significant obstruction or thrombus (Figure 8).

Considering the main cause of large cardiac dilatations is aortic valve insufficiency,^21^ it is important to highlight that, in this patient, there was no such dysfunction. On the other hand, there was insufficiency of both atrioventricular valves, possibly as a result of dilatations and valve rings. In the other organs, there were signs of severe congestive heart failure, with chronic passive congestion of the lungs and liver and acute lung edema, which led to cardiogenic shock, with prerenal acute renal failure (because there was no acute tubular necrosis) and hepatocyte necrosis in the centrilobular region. Shock was the final factor that lead to the patient's death.

The patient had a history of bronchopneumonia, which must have been adequately treated, because, during the necropsy, there were only focal areas with compatible aspects with suspected resolved bronchopneumonia.

**(Dr. Paulo Sampaio Gutierrez)**

## Comment

The main question brought by this case is the difficulty, even with the necropsy, of establishing a differential diagnosis between ischemic heart disease and idiopathic DCM. The dilatation is enormous, with a weight over 1,000 grams.^21^ There is no aortic insufficiency. Morphological pattern of the myocardium is, to a certain extent, indicative of ischemia, with fibrosis and areas of necrosis in organization, with a tendency to be transmural in the diaphragmatic wall, as shown by the MRI and the necropsy. The aspect is compatible to a healed myocardial infarction. The other areas of ischemia may be secondary to the heart failure caused by such infarction. On the other hand, coronary arteries do not have any obstructive lesion; thus, the case can also fit as a case of DCM, with the necrosis, in this case, also being secondary to the increase in the myocardial mass. Considering the absence of coronary obstruction and the fibroses pattern that is not completely transmural, this last possibility may seem the most adequate. To explain it, even without excluding the possibility that it might be idiopathic, corresponding to a myocarditis that did not evolve or to spontaneous vasospasm, we must consider the hypothesis of the drug use. The patient reported to have been a cocaine user, even if in the past, and methylenedioxymethamphetamine ('ecstasy') was found in his blood. There are reports that both drugs can cause infarction by vasospasm or even fixed obstructive lesions^22,23^ and have also been associated to DCM.^24^

**(Dr. Paulo Sampaio Gutierrez)**

**Main disease:** DCM (though we cannot completely rule out the possibility of an ischemic disease), possibly related to drug use.

**Cause of death:** cardiogenic shock.

**(Dr. Paulo Sampaio Gutierrez)**

**Editor da Seção:** Alfredo José Mansur (ajmansur@incor.usp.br)

**Editores Associados:** Desidério Favarato (dclfavarato@incor.usp.br)

Vera Demarchi Aiello (anpvera@incor.usp.br)
